# Supplementary material for: Genome-Wide Association Study and Genomic Prediction of Fusarium Wilt Resistance in Common Bean Core Collection
Source: Int J Mol Sci. 2023 Oct 18;24(20):15300. doi: 10.3390/ijms242015300 (PMC10607830; doi:10.3390/ijms242015300)
Supplement: Supplementary file 1 [file ijms-24-15300-s001.zip › Supplementary fig S1-S8.pdf]

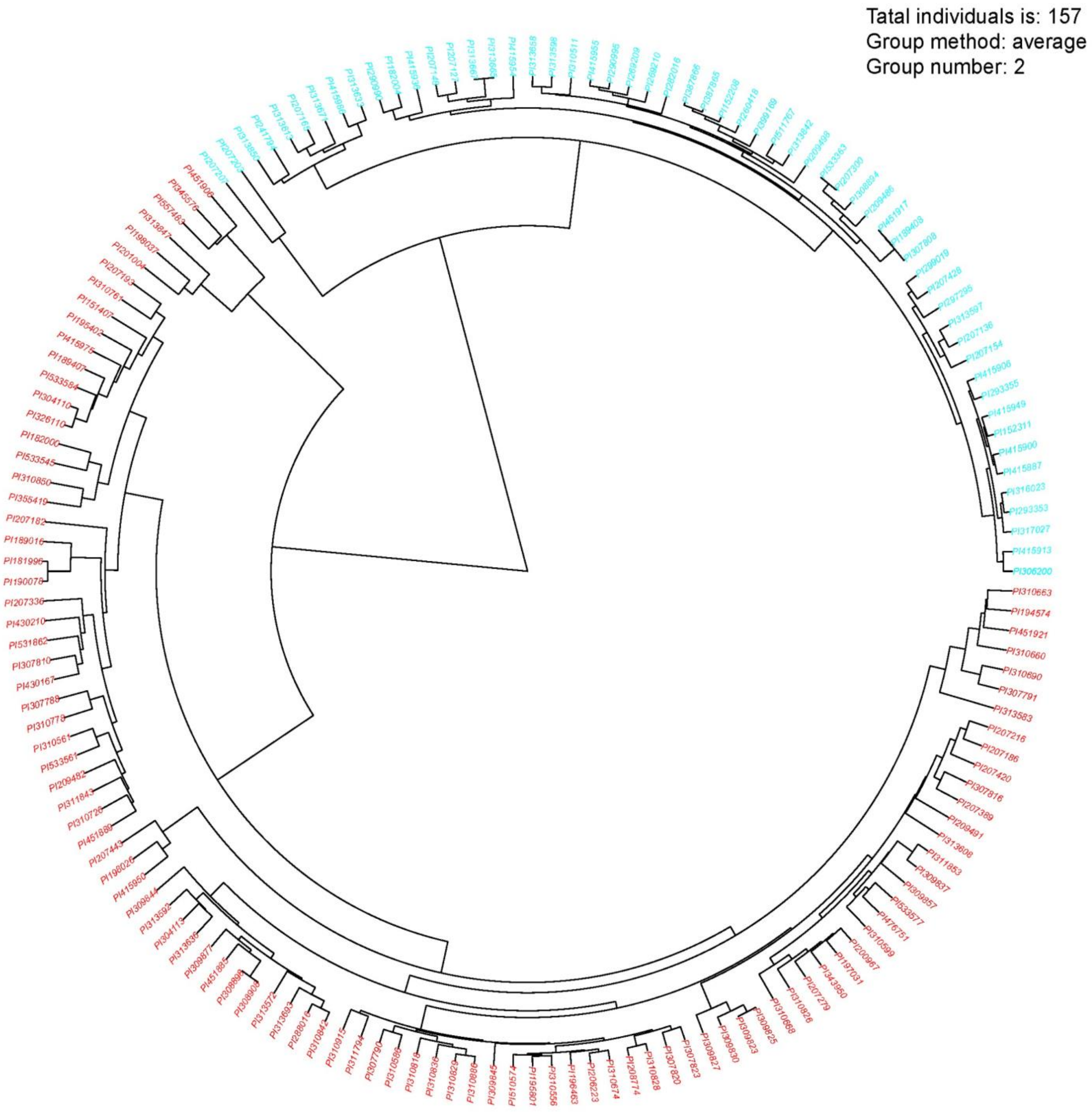

Figure S1: Phylogenetic tree of two sub-populations by neighbor-joining (NJ) method drawn by GAPIT 3 in 157 common bean accessions.

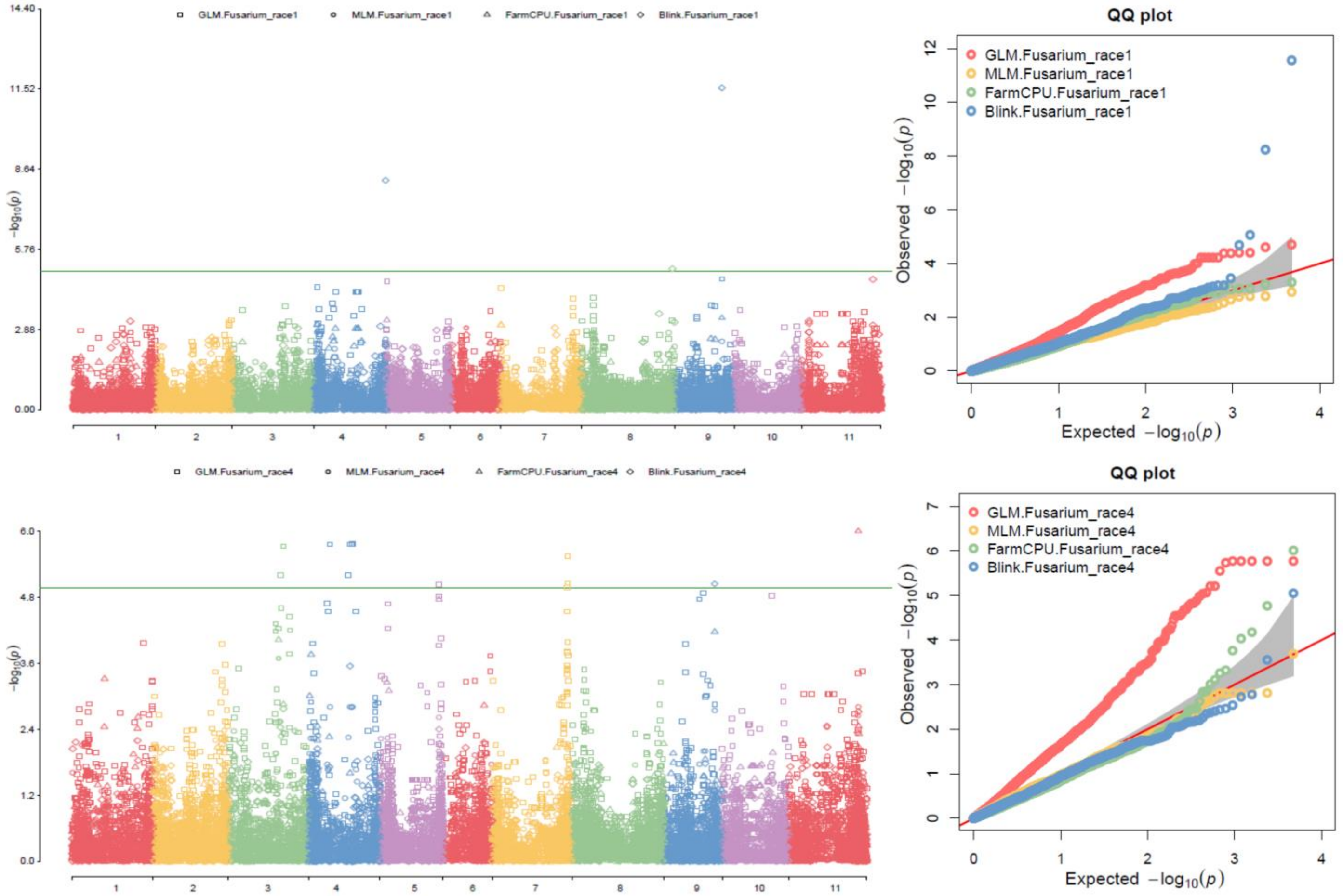

Supplementary Figure S2: The multiple Manhattan and QQ symphysic plots of four models, GLM, MLM, FarmCPU, and BLINK for re-sistance to Fusarium wilt (race 1 (top) and race 4 (bottom)) by GAPIT 3.

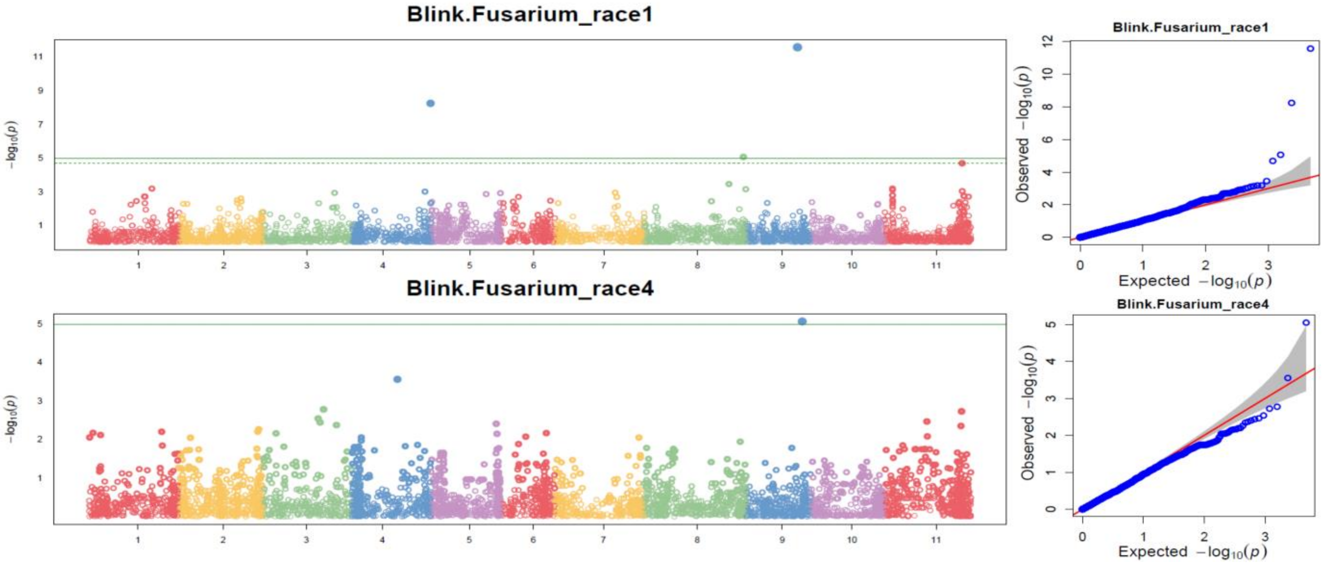

Supplementary Figure S3: The Manhattan and QQ plots of BLINK model for resistance to Fusarium wilt race 1 and race 4 by GAPIT 3.

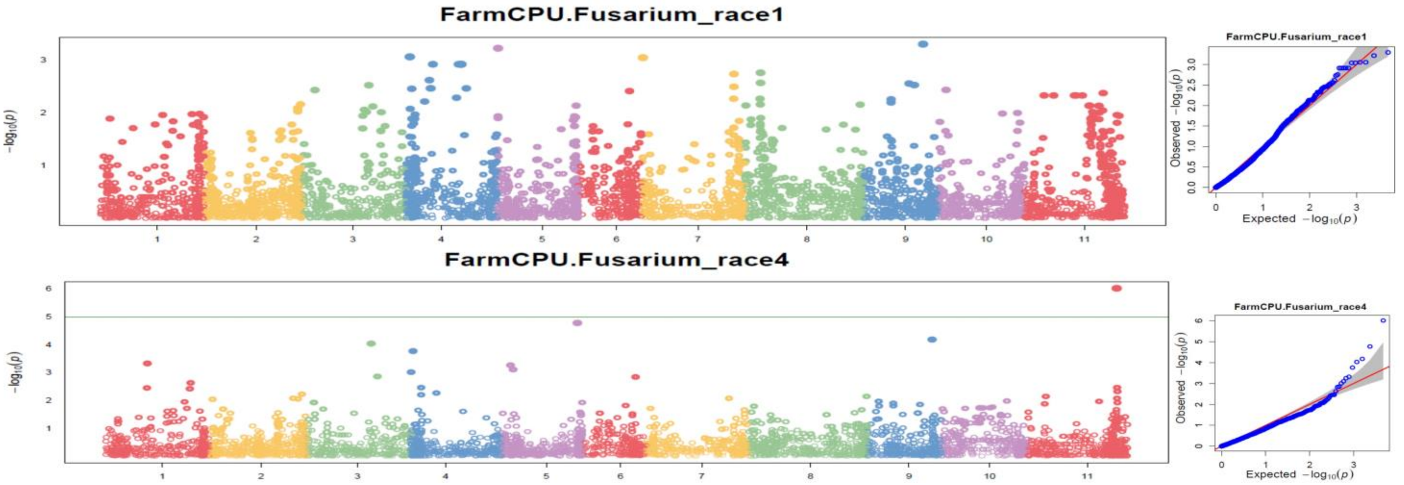

Supplementary Figure S4: The Manhattan and QQ plots of FarmCPU model for resistance to Fusarium wilt (FW) race 1 and race 4 by GAPIT 3.

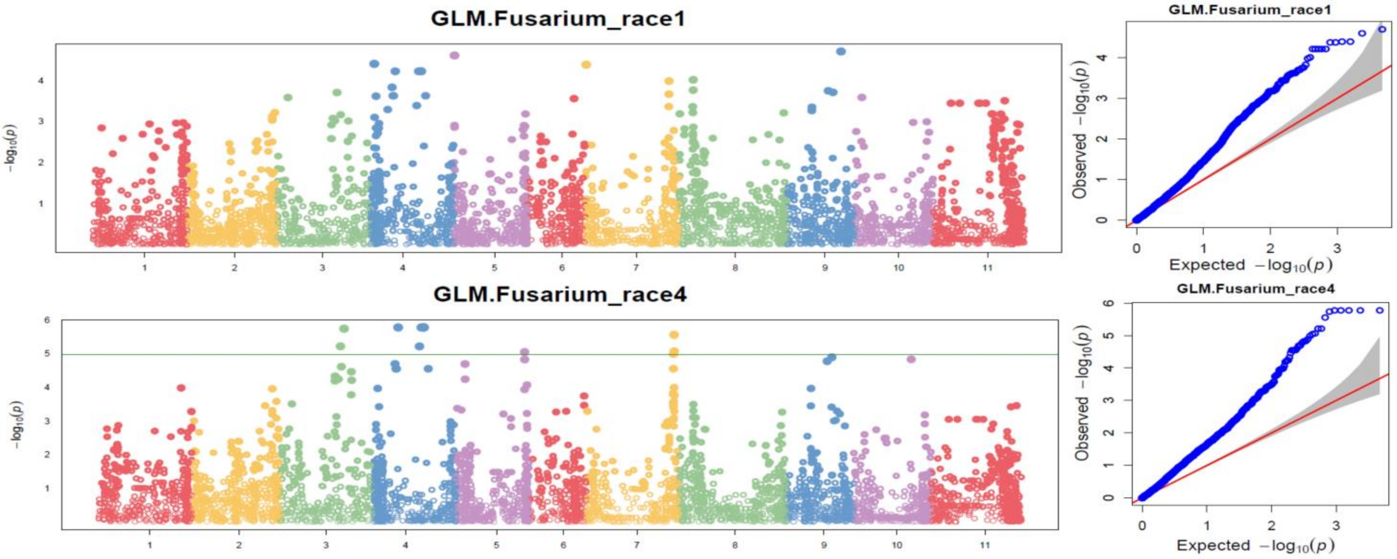

Supplementary Figure S5: The Manhattan and QQ plots of GLM model for resistance to Fusarium wilt (FW) race 1 and race 4 by GAPIT 3.

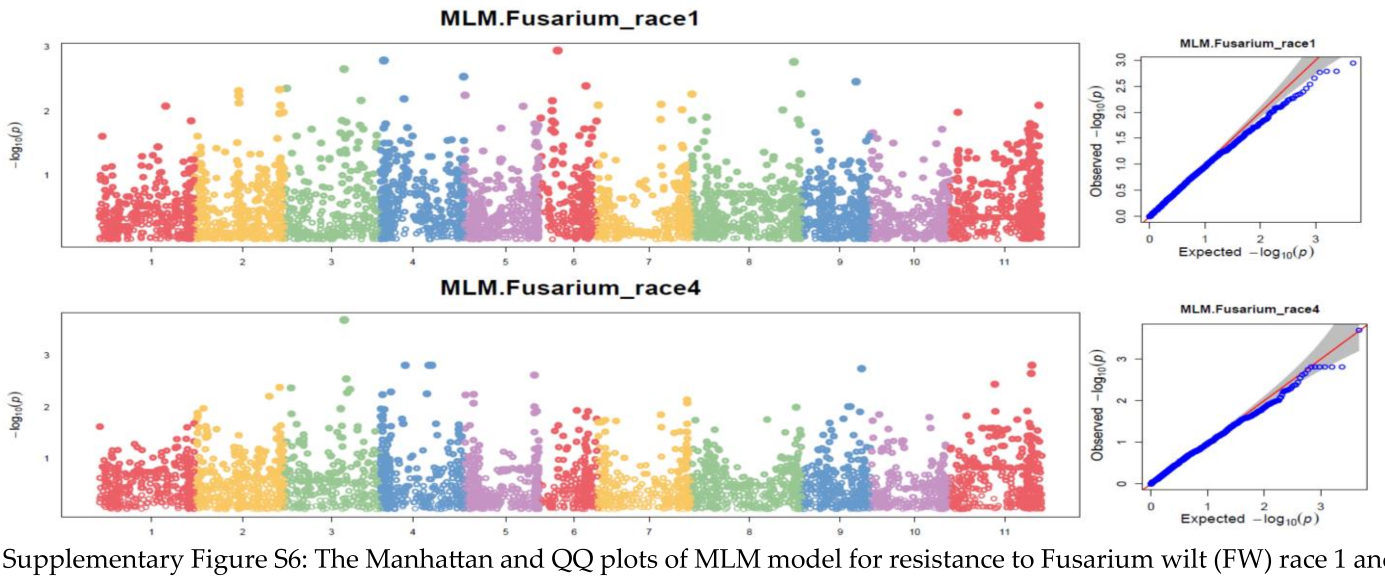

Supplementary Figure S6: The Manhattan and QQ plots of MLM model for resistance to Fusarium wilt (FW) race 1 and race 4 by GAPIT 3.

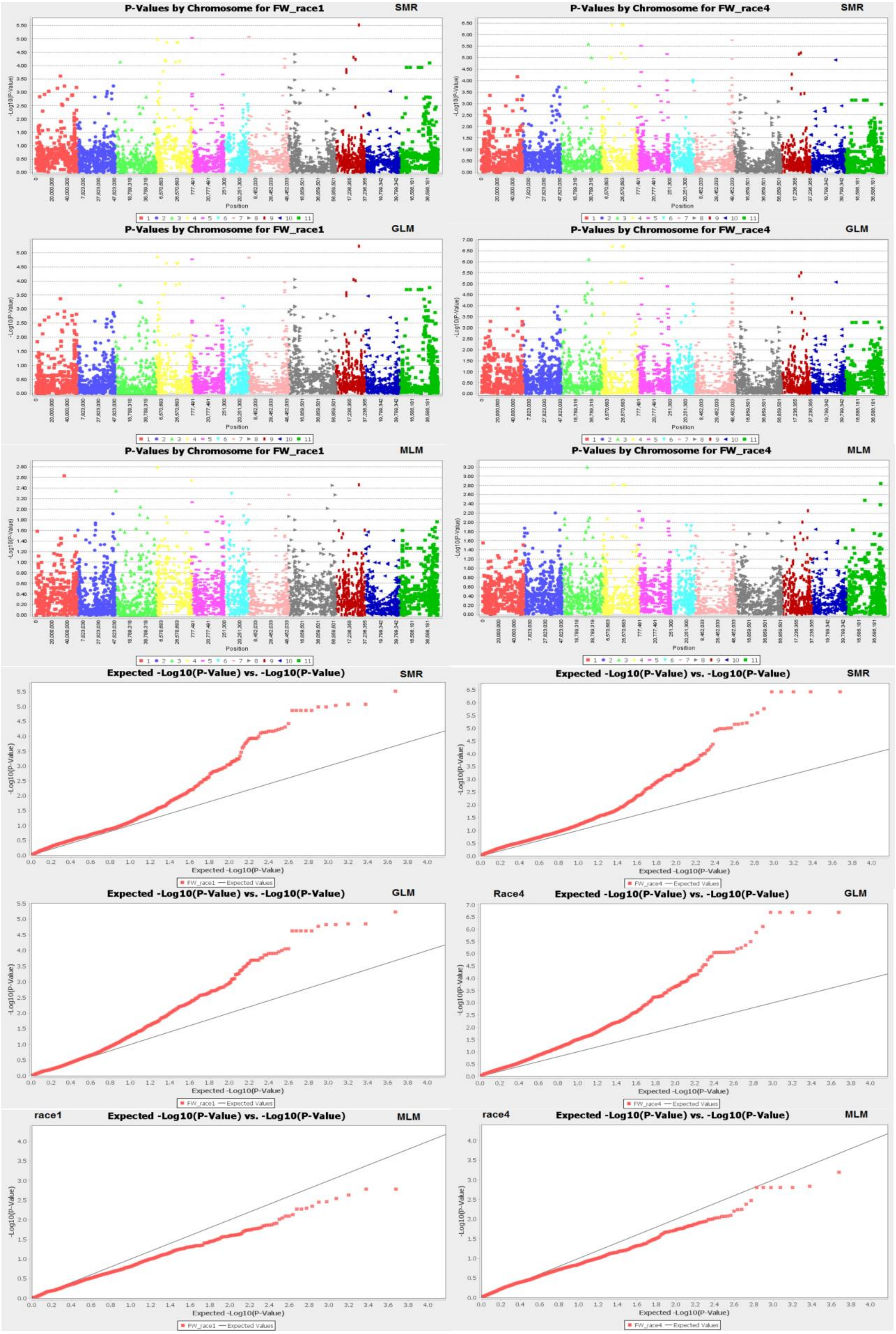

Supplementary Figure S7: The Manhattan and QQ plots of SMR, GLM, and MLM models for resistance to Fusarium wilt (FW) race 1 and race 4 by TASSEL 5.

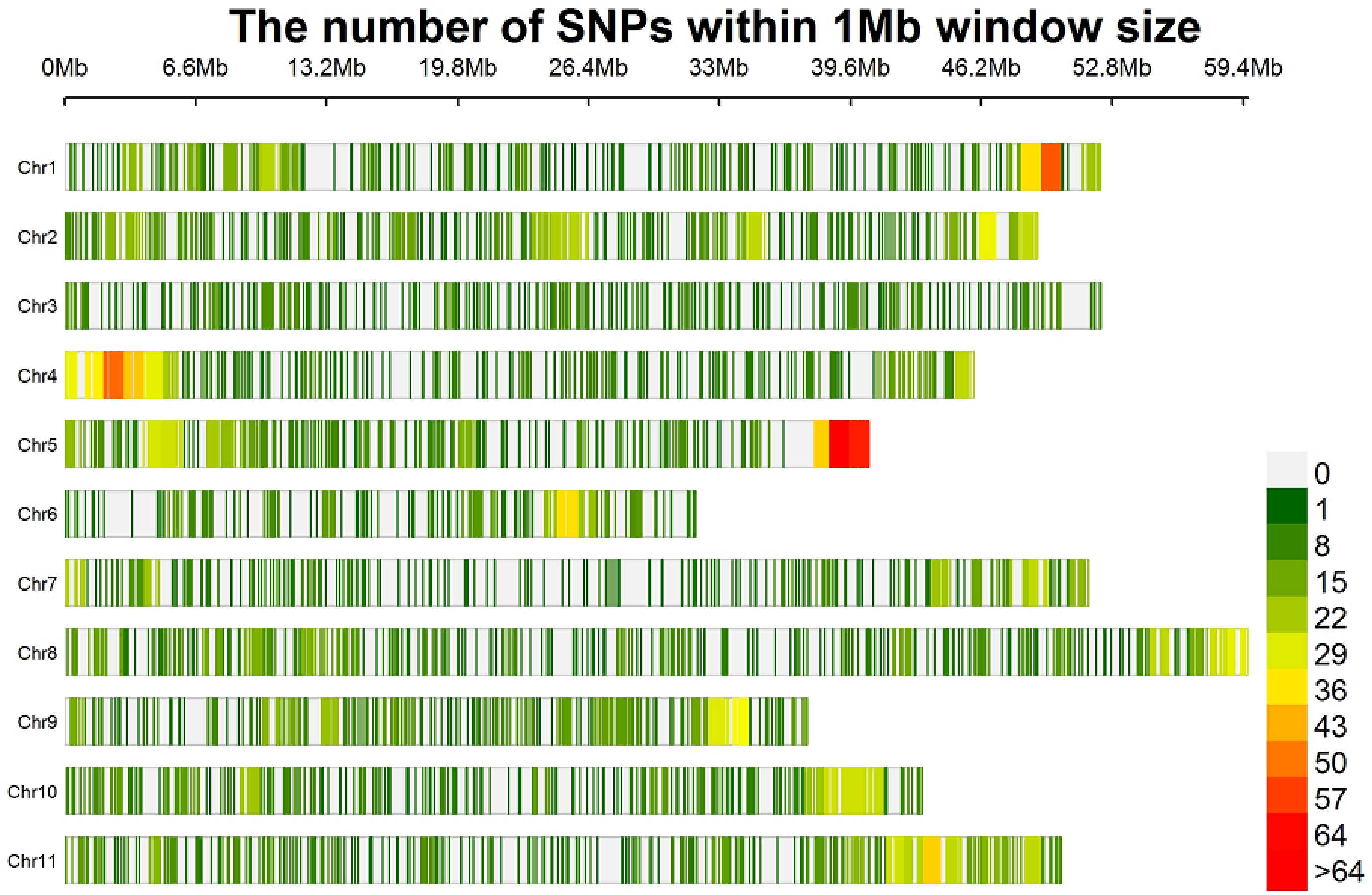

Supplementary Figure S8: Distribution of the 4740 SNP on the 11 chromosomes of common bean. Chromosomes are on the vertical axis. Chromosome length in Mb is on the horizontal axis, and the color represents the number of SNPs per 1 Mb window size, SNP density.
